# Preliminary evidence of linguistic bias in academic reviewing

**DOI:** 10.1016/j.jeap.2020.100895

**Published:** 2020-07-28

**Authors:** Stephen Politzer-Ahles, Teresa Girolamo, Samantha Ghali

**Affiliations:** a The Hong Kong Polytechnic University, Hong Kong; b University of Kansas, USA

**Keywords:** Linguistic injustice, Academic publishing, Implicit bias, Peer review

## Abstract

Recent years have seen a spirited debate over whether there is linguistic injustice in academic publishing. One way that linguistic injustice might occur is if gatekeepers (e.g., peer reviewers and editors) judge the scholarly quality of academic writing more harshly if the writing does not meet expectations for international academic English, even if the content is good. We tested this with a randomized control study in which scholars judged the scientific quality of several scientific abstracts. Each abstract had two versions with identical scientific content, such that the language in one version conformed to standards for international academic English, and the language in the other version did not (but was still comprehensible). While the data are preliminary and the effects statistically inconclusive, both pre-registered and exploratory analyses of the data suggest that scholars may give abstracts lower ratings of scientific quality when the writing does not conform to standards of international academic English. These results suggest that linguistic bias may occur in academic peer reviewing and motivate further study to better understand and address this phenomenon.

## 1. Introduction

Do scholars who work and publish in a second language (typically English) that is not their dominant language encounter linguistic prejudice or injustice? That is to say, do language-related challenges or bias make it more difficult for them to engage in scholarship or disseminate their work, compared to scholars for whom English is their dominant language and who learned English from birth? Non-native speaker disadvantage is a topic that has been covered widely by scholars in English for research publication purposes. Previous research on the difficulty of publishing in English as a second language by non-native speakers of English (e.g., Englander, 2009; Hanauer & Englander, 2011), pressure on multilingual scholars to publish in English (e.g., Curry & Lillis, 2004; Lillis & Curry, 2006), and the politics of knowledge dissemination in English (e.g., Corcoran & Englander, 2016) has informed the concept of linguistic disadvantage. These studies generally report ways in which scholars who publish in a second language face burdens that scholars publishing in English as their first language do not face or face to a lesser extent.

More recently, however, the notion that scholars publishing in a second language face unique challenges and biases has come under question. In an influential paper, Hyland (2016a) challenged this orthodoxy by pointing out that there was not yet any convincing direct evidence that such injustice occurs in academic publishing, and that assuming that there are such challenges for scholars working in a second language may both send discouraging messages to such scholars and may distract from more important injustices (such as geographic inequities in access to research resources). This paper kicked off several years of lively debate, which is ongoing. Politzer-Ahles, Holliday, Girolamo, Spychalska, and Berkson (2016) argued that it is likely that linguistic injustice does occur and that a lack of positive evidence did not constitute a lack of linguistic bias itself. In a response to this response, Hyland (2016b) highlighted the need for direct evidence, rather than speculation.

In the years since this series of papers issued this challenge to the field, several more papers have put forth conceptual arguments for or against the existence of linguistic injustice. For example, Flowerdew (2019) and Yen and Hung (2019) argued that linguistic injustice in academic publishing does exist and is worth attention. Conversely, Hultgren (2019) agreed with Hyland (2016a,b) that the idea of linguistic injustice detracts from more important injustices in academic publishing and, at worst, borders on language policing or “verbal hygiene”. Perhaps more importantly, given the stated need for empirical data, several recent studies have offered observational data that supports or challenges the claim that there is linguistic injustice (Table 1 offers a rough summary of the most relevant studies that we are aware of). To our knowledge, however, no study has yet reported the sort of controlled experimental manipulation that would provide the strongest evidence for or against the existence of linguistic bias.

## 2. The need for experimental studies

While the studies in Table 1 have employed both quantitative and qualitative methods, all were observational. An observational study may well reveal data (whether through quantitative results like numbers of publications, or qualitative patterns like authors’ and reviewers’ self-reported experiences and views) suggesting that scholars for whom English is a second (or third, fourth, etc.) and non-dominant language face relatively more difficulty compared to scholars for whom English is a first, and dominant, language. However, an observational study is incapable of ruling out potential non-linguistic factors. For example, it is an open question to what extent the disproportionately lower number of publications from the former group may be due to a lack of institutional resources, rather than linguistic prejudice. Similarly, it is not known to what extent authors may perceive second-language publishing as difficult not because of actual difficulty, but because a traditional perspective that focuses on nonnative-ness as a “deficit” may discourage scholars working in a second language and train them to perceive working in that language as difficult. Authors’ subjective perception of difficulty, as self-reported in interviews or surveys, may not necessarily translate into a reality of less publication success (see, e.g., Hultgren, 2019). (Our meaning here is not to blame such scholars for difficulties they may encounter, but to acknowledge the potential harms of a “deficit” view of scholars working in a second language—an issue discussed at greater length by Hultgren, 2019, among others.) Politzer-Ahles et al. (2016) suggested that one way to empirically test for evidence of one kind of linguistic injustice would be through a randomized control trial, rather than by collecting observational data in which language background and adherence to conventions of international academic English are inextricably confounded with many other factors.

Specifically, in their proposal, Politzer-Ahles et al. (2016) focused on one specific way in which linguistic injustice may manifest: the possibility that peer reviewers judge academic papers more negatively if the language does not conform to expected international academic English standards, regardless of the quality of scholarship. Here we operationally define alignment to international academic English standards to be roughly equivalent to what academic reviewers with no special training or expertise in the use of English as a lingua franca or additional language consider to be “good” and “native-like.” If two papers represent equally “good” research, but one is written in English that conforms to what the reviewer considers to be “good” English and another paper is not (but is still just as comprehensible), will a reviewer be more likely to recommend publication of the first paper than the second? If so, that would be a concrete manifestation of linguistic injustice. Hyland (2016a) argued that reviewers do not do this—specifically, that reviewers are able to separate linguistic factors from judgments of scholarly quality, and that papers generally do not get rejected because of language alone. However, Strauss (2019) reported that reviewers do appear to carry language-based assumptions and biases. Of course, biases are often subconscious, such that people may very well be unaware of their own biases (Banaji & Greenwald, 2013). Thus, the question of whether biases about language influence reviewer judgments of manuscripts is one that ought to be tested experimentally, rather than through introspection or interviews. This is what Politzer-Ahles et al. (2016) proposed as a potentially fruitful approach to testing whether one form of linguistic injustice occurs.

Fortunately, experimental methods already exist for testing implicit biases. In the most famous example, Bertrand and Mullainathan (2004) sent employers in the United States otherwise identical resumes that had either names commonly associated with Black people (e.g., Lakisha or Jamal) or names commonly associated with white people (e.g., Emily or Greg). Employers were less likely to call back the fake applicants with Black-sounding names than those with white-sounding names, even though their application materials were otherwise exactly the same. While this particular manipulation confounded perceived race with perceived socio-economic status (Simonsohn, 2015, 2016) and names may not be the most controlled way to manipulate social biases, the study nevertheless demonstrated how to manipulate one factor (i.e., the name written on the resume) while holding other factors constant. Comparing success in job applications between individuals with different races or socioeconomic status in observational data would make it difficult to isolate effects of these factors, since people of different races or socioeconomic statuses may often have very different resumes, because of widespread differences in access to education, social capital, and other resources. On the other hand, conducting an experiment with fake job applications gives researchers complete control over the content of these experiment materials.

This kind of experiment design has been applied to academic contexts as well. Moss-Racusin, Dovidio, Brescoll, Graham, and Handelsman (2012) had science faculty evaluate fake job applications for research assistant positions, and found that fake applicants perceived to be men were considered more competent and hirable than otherwise identical applicants perceived to be women. Knobloch-Westerwick, Glynn, and Huge (2013) had scholars evaluate fake research abstracts, and found that in certain fields, abstracts written by authors perceived to be men were considered to have greater scientific quality than otherwise identical abstracts written by authors perceived to be women.

Additionally, vignettes, or written information in a narrative paragraph format, have been found to be a useful and cost-effective methodology to evaluate practitioner decision-making. Previous studies have used vignettes to determine how teachers perceive and make educational decisions for children with Attention Deficit/Hyperactivity Disorder (Egyed & Short, 2006; Moldavsky, Gorenewald, Owen, & Sayal, 2013), as well as how speech-language pathologists’ clinical decision-making for children with limited linguistic competency (Selin, Rice, Girolamo, & Wang, 2019). In addition to education and communication science and disorders, medicine has also used vignettes to assess practitioner decision-making and found that the quality of care (i.e., the series of decision-making that led to improved outcomes) from vignettes approximated that from standardized patients (i.e., the gold standard in medicine; Dresselhaus, Peabody, Luck, & Bertenthal, 2004; Peabody, Luck, Glassman, Dresselhaus, & Lee, 2000).

We provide here a brief overview of vignettes using an example from Selin et al. (2019). Speech-language pathologists read a sequence of 6 vignettes describing the profile (i.e., classroom behavior) of a child with limited linguistic competency. For example, “S. C. is a 7-year 5-month-old boy who was referred for speech-language pathology services by his teacher. His teacher stated that S. C. got along well with his classmates although he was somewhat shy … “ (pp. 306). The characteristics of each profile were systematically varied along multiple dimensions, such as language impairment status (impaired, borderline impaired, or typically developing). After reading each vignette, respondents answered a series of items indicating what clinical decisions they would make (i.e., recommendation of services and types of specific services), as well as what they saw as areas in need of improvement (e.g., pragmatics, syntax). Critically, respondents were instructed to use only the criteria in the vignettes to answer items and could not return to a vignette after completing the items. These conditions - disallowing the ability to post-hoc change item responses and providing explicit instruction on what to evaluate - maximized the likelihood of reflecting the “true” perception of speech-language pathologists and how this informed their decision-making responses.

Thus, vignettes are an informative method that may be equally informative for understanding the reviewal of academic abstracts for conferences. Just as practitioners from these fields, reviewers for academic conferences are in the explicit role of assessing academic work and making decisions that have real-world impact. That is, reviewer decision-making helps determine what science will be presented at a scholarly meeting and will ultimately help progress the field. Therefore, it is important to examine how people who serve as reviewers for academic conferences may perceive differences between abstracts that vary along one parameter: whether or not they conform to the conventions of international academic English.

## 3. The present study

The goal of the present study was to use the same approach to test whether peers consider scientific writing to have lower scientific quality if it is written in English that does not conform to international academic English standards.

We introduced this question above by asking if two pieces of equally “good” research would be judged differently based on how “good” their English writing is. To test this, we must define what constitutes “good” research, as well as what constitutes “good” English. Research quality might refer to the methodological rigour of the study conducted, to the extent that the conclusions are justified (i.e., a paper that reports a not-so-rigorous study but then makes nuanced conclusions that show awareness of the limitations may be “better” than a paper that reports conducts a methodologically rigorous study but then oversells the conclusions that can be drawn from it), to the perceived novelty or impact of the work, or to any combination of these or other factors. In the present study we only asked respondents to rate the “scientific quality” of each work, without giving explicit instructions on what dimension of scientific quality they should evaluate. Further, we did not provide information on the linguistic background of abstract authors. Thus, the assumption was that respondents could focus on any combination of the above dimensions or other dimensions we have not described.

What about what constitutes “good” English? Here we employ scare quotes in acknowledgement of the fact that this is a term that is not useful and is not used in discussion of English as an additional language or English for academic research publication purposes. When people colloquially refer to “good” English, they generally are referring to English that conforms to international standards of what English written by educated native speakers of a mainstream English variety (typically American or United Kingdom) is expected to look like in terms of grammar and register. These standards include prescriptive grammar rules (e.g., subject-verb agreement, how articles should be used, etc.), as well as stylistic and register expectations about how academic writing normally looks. In our case, we wanted to test whether reviewers would change their judgment of scientific quality based only on how well the language conformed to these English standards. Of course, if the English deviated so far from these standards that the reader could not understand the procedure of the study or the claims of the authors, then this could influence their judgment of scientific quality for reasons not directly related to language bias. That is not the case we were interested in testing. Our interest was in whether reviewer judgment of scientific quality would be influenced by language mechanics even if their ability to understand the work was unimpaired. Thus, in the abstracts we used for the present study, we manipulated the adherence of the writing to prescriptive grammatical forms, rather than the adherence of the writing to register and stylistic expectations.

We prepared four short abstracts describing research in communication science and disorders (based on real abstracts provided by colleagues), and edited each abstract to have one version with “international academic standard” English (hereafter referred to as “standard English” for brevity) and one with English not conforming to international academic standard English conventions (hereafter referred to as “non-standard English” for brevity). The example below shows both versions of the opening sentence of one abstract; the two versions of the abstract from which these examples were taken are included in the Appendices, and the complete set of materials is available at https://osf.io/mwd6z/ (in a folder labelled “Materials”).

1a. **“standard” English:** *While much research has focused on first language acquisition of tone, there is little study of the development of tone in bilingual children whose dominant language is non-tonal and who are exposed to a tone language in school.*
1b. **“non-standard” English:** *While much of the research focused on first language acquisition of tone, there is little study of development of the tone in bilingual child whose dominant language is non-tonal and who are exposed to tone language in school.*

As the example demonstrates, the scientific content was identical, and the sentence should be easy to understand in either version. The English in the second example included several patterns which are common in English academic writing by native speakers of some other languages (for example, different use of tense/aspect [compare “has focused” vs. “focused”], different use of “the” [compare “development of tone” vs. “development of the tone”], and more). Our goal was for readers of each abstract to be able to understand the content equally well. While it was possible that the writing differences may still have influenced comprehension (e.g., someone reading “study on the development of the tone” could misunderstand this as referring to the development of one particular tone), we felt that this manipulation kept the clarity of the content as similar as possible between the two abstracts while still keeping the language differences easily noticeable.

We recruited scholars in communication science and disorders to evaluate these abstracts as if they were reviewing them for a conference. We predicted that, if academic reviewers’ judgments of scientific quality were biased by their judgment of language mechanics, they would give lower ratings of scientific quality to abstracts written in “non-standard” English. This would constitute evidence for one kind of linguistic injustice.

Here a note about terminology is in order. When we refer to “standard” and “non-standard” patterns of usage, we are referring to how these patterns may be perceived by readers, not to where these patterns actually come from. We are under no illusions that all “native” English speakers write at what reviewers would consider an ideal standard, nor that all “non-native” speakers write below that standard. Furthermore, we are not suggesting here that all, or even most, “non-native” speakers write in the way exemplified in our “non-standard” example. We specifically edited these abstracts to have “worse” English (i.e., English that is less consistent with what readers expect international-standard academic English to look like) than that of most “non-native” English-speaking scholars we have worked with or read. Our purpose is not to claim that all “native” speakers write one way and all non-native speakers write another way; indeed, many authors have challenged the utility of the notion of native-ness when discussing English for academic purposes (e.g., Hultgren, 2019), and have highlighted that the register and style of academic English is not really anyone’s native language (e.g., Hyland, 2016a). Rather, our purpose was to see how reviewers reacted when reading text that *they* may associate with native-like-ness/standard-ness or non-native-like-ness/non-standard-ness. We aimed to investigate biases against aspects of language, not biases against people. Hence, throughout this paper, we use the terms “standard” and “non-standard” in scare quotes, to acknowledge that not all scholars who learned English in adulthood write in “non-standard” academic English, and not all scholars who learned English from infancy as their dominant language write in “standard” academic English. If one adopts the additional assumption that scholars who learned English later in life have to work more to attain “standard” English academic writing proficiency than scholars who speak English as a first and dominant language do, then a finding of bias against “non-standard” academic English in the present study would entail greater challenges for non-native English speakers than native English speakers.

As the present study was a preliminary investigation, there were many aspects left open for further study. For example, it would be worthwhile to evaluate abstracts in a real context by actually submitting abstracts to a conference, rather than by sending them out in a survey. Such methods would provide additional ecological validity. We designed this study as an initial exploration into whether this approach to testing linguistic bias is worth pursuing.

## 4. Methods

Methods for the experiment were pre-registered at https://osf.io/bngfj.^1^ Any analyses that deviate from the pre-registration are reported here as exploratory.

Methods were approved by the Human Subjects Ethics Sub-Committee at The Hong Kong Polytechnic University (reference: HSEARS20161219001).

### 4.1. Participants

We recruited participants through e-mail discussion lists and online forums for scholars involved in psycholinguistics (CUNY Conference on Human Sentence Processing, Architectures and Mechanisms for Language Processing), communication science and disorders/speech-language-hearing sciences (Asian & Pacific Islander Speech-Language-Hearing Caucus, the Minority Student Leadership Program of the American Speech-Language-Hearing Association, and the American Speech-Language-Hearing Association itself, as well as two of its student interest groups, the National Student Speech-Language-Hearing Association and Mentoring Academic-Research Careers), and the cognitive sciences (SPARK Society). Demographic information from the respondents is available at https://osf.io/mwd6z/. We closed the survey when we had responses from 102 respondents. The first six respondents completed a version of the survey with an error which prevented the abstracts from being displayed, and thus are not analyzed. After closing the survey and viewing the data, we discovered that many participants did not complete the entire survey and did not respond to all (or even any) of the abstracts. Overall, 37 respondents completed the survey (responding to all abstracts), an additional 16 respondents provided partial responses (responding to some, but not all, of the abstracts), and 43 did not evaluate any of the abstracts.

### 4.2. Materials

Colleagues who volunteered to have their abstracts used in this project provided four real abstracts. We edited the abstracts to roughly mimic the format of the American Speech-Language-Hearing Association Convention. As such, each abstract was 200e300 words in length and divided into four sections: “Rationale”, “Method”, “Results”, and “Discussion”. The abstracts described studies about acquisition of lexical tone in bilingual children, language assessment in bilingual children, motor memory of Chinese characters in a post-stroke client, and speed of Chinese character writing. Furthermore, the authors edited each abstract to conform to international-standard academic English. To make “non-standard” versions of each abstract, one native Chinese speaker translated the abstracts into Chinese, another native Chinese speaker translated them back into English, and we further edited the abstracts to ensure that the scientific content remained identical and comprehensible across abstracts (e.g., to correct any misstatements about the research that arose in the translation process) and to make sure that the writing clearly did not conform to international-standard academic English but was still comprehensible. The full abstracts are available at https://osf.io/mwd6z/.

### 4.3. Procedure

Participants completed a survey with the abstracts on Qualtrics, an online survey platform, to isolate the role of reviewer behavior, the survey did not provide information on the linguistic background (i.e., native-like versus non-native like academic English) of the abstract authors. As such, participants reviewed each abstract blind to the linguistic background of all authors, thus increasing the likelihood of revealing linguistic bias. After providing informed consent, participants responded to questions in major content blocks: 1) demographics (race/ethnicity and sex); 2) language background (languages spoken and proficiency in each language); 3) professional background (current academic/career position, duties, proportion of people at their institution and discipline speak their languages, frequency of communication with fellow academics in a language other than their first language); and 4) abstracts. The questions in the first three blocks were included to facilitate later exploration and were unrelated to the pre-registered analysis plan. After completing blocks 1–3, participants read the abstracts one at a time. For each of the four abstracts, they rated the scientific quality on a five-point ordinal scale (“Fundamentally unacceptable”, “Poor”, “Fair”, “Hiqh quality research”, “Top level of research”) and could select zero or more areas in need of improvement (“Scientific content”, “Scientific novelty”, “Strength of findings”, “Other” [with a text input field], and “Nothing”). Participants also rated the clarity of the abstract on a five-point ordinal scale (“Incomprehensible”, “Difficult to understand”, “Somewhat clear”, “Mostly clear”, “Completely clear”) and selected areas for improvement (“Formatting”, “Language (i.e., syntax, grammar, vocabulary)”, “Organization”, “Writing style”, “Other” [with a text input field], and “Nothing”). Finally, participants had the option to provide further feedback (i.e., write comments to the author). Upon completion of the last block (i.e., the abstracts), the participants responded to an item asking whether they are an editor or reviewer for an academic journal published in English, and indicated their exact role as a reviewer.

The abstracts were meant to be arranged into two lists in a Latin square design, such that in the first list (which would be seen by half of the participants), abstracts #1 and #3 would appear in the “non-native-like English” condition and abstracts #2 and #4 would appear in the “native-like English” condition, whereas in the second list (which would be seen by the other half of the participants), the mapping between abstracts and conditions would be switched. The pre-registered analysis plan assumed this arrangement. Due to an error in the online survey setup, the abstracts were not presented in this way: half of participants saw the first list as planned, whereas the other half saw all four abstracts in the “standard English” condition. In other words, abstracts #1 and #3 were seen in the two different conditions (by different participants), whereas abstracts #2 and #4 were always seen in the “standard English” condition. Below we report additional exploratory analyses that take this limitation into account.

### 4.4. Analysis

The only dependent variable analyzed was the five-point rating of scientific quality. We regressed this ordinal quality rating on the language condition (“native-like” English vs. “non-native-like” English) using a cumulative link mixed effects model. The pre-registered analysis plan also included List as another fixed effect in the model, interacting with language condition; however, we could not include this interaction because of the failure to properly present the second list, as explained above. For this mixed-effect model analysis we included all ratings we received, including those from participants who did not rate all four abstracts.

Below we also report several alternative, exploratory analyses, given that the data format we received differed in several ways (missing data from many participants, lack of a List 2) from what we had expected when we pre-registered the analysis plan.

## 5. Results

All data and statistical analysis code are available at https://osf.io/mwd6z/.

### 5.1. Pre-registered analysis

**Fig. 1.**
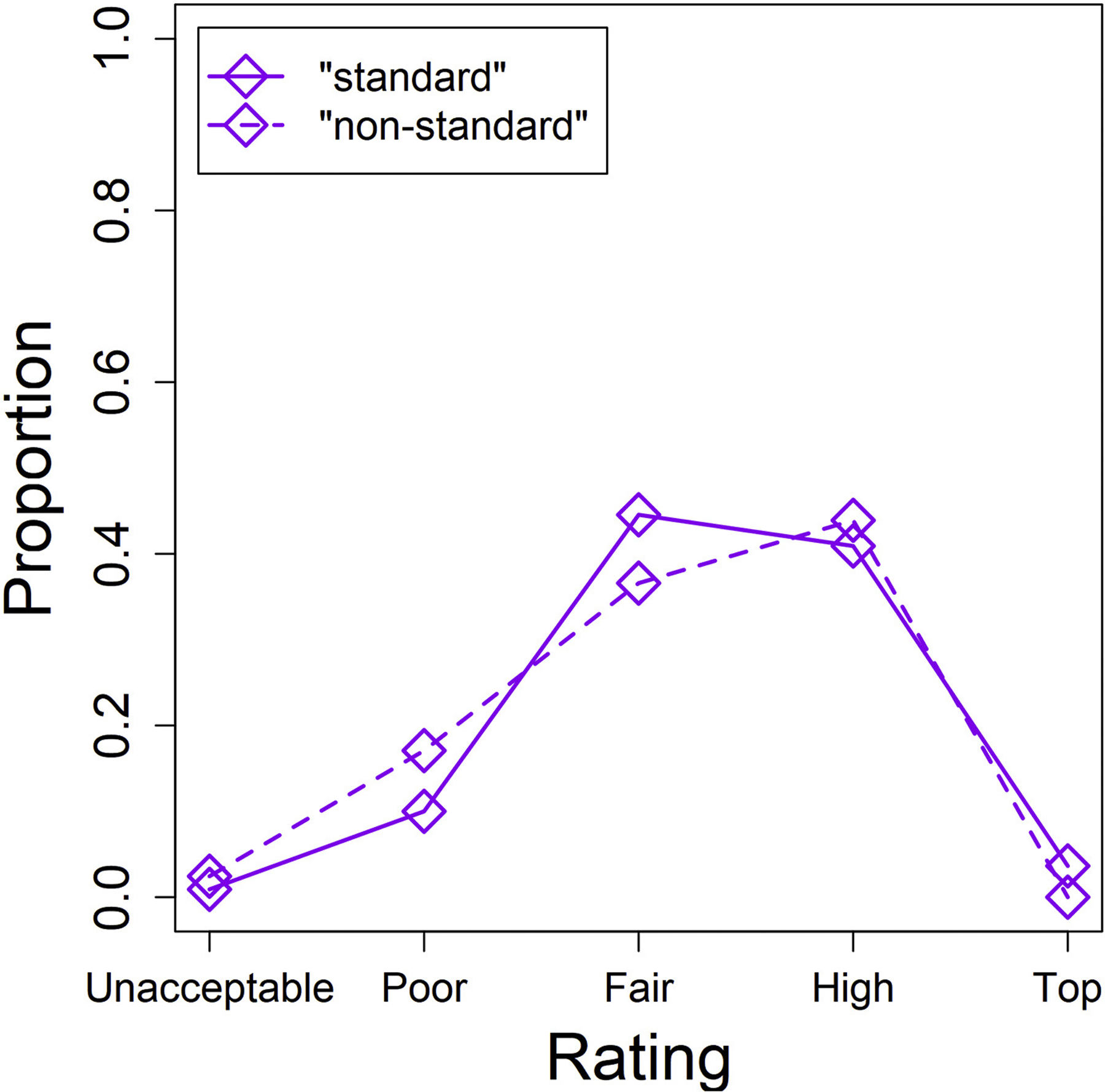
Proportion of participants giving each rating to the “standard English” abstracts (solid line) and to the “non-standard English” abstracts (dashed line).

Fig. 1 shows the proportion of each rating for each abstract condition.

“Non-standard English” abstracts were much more likely than “standard English” abstracts to be labelled “Poor” and much less likely to be labelled “Fair”, indicating that at this relatively low quality level, abstracts perceived as having “better” English (more consistent with conventions of international academic English) tended to be rated more highly. “Non-standard English” abstracts were slightly more likely than “standard English” abstracts to be rated “High quality” and slightly less likely to be rated “Top level”, although these differences are quite small compared to the abovementioned differences.

The statistical model showed that abstracts with “non-standard English” received lower ratings than abstracts with “standard English” (*b* = −1.34, *z* = −1.97, *p* = .048).

### 5.2. Exploratory analyses

Because of missing cases and missing conditions in the data (see Methods section), the pre-registered analysis may have been less appropriate than it would have been if we had collected all the data we intended. For example, attempting to model random slopes of language condition for participants and abstracts may not make sense when some participants and abstracts did not have observations in both language conditions. Therefore, here we present several exploratory analyses treating the data in different ways. As these are exploratory, the *p*-values should not be used to justify any strong interpretations. However, these analyses may provide some clues as to whether there are trends worth investigating with new data in the future.

First, we removed participants who did not rate all abstracts, and re-ran the pre-registered model on this subset of the data. The qualitative pattern was in the same direction, but not statistically significant (*b* = −0.94, *z* = −1.33, *p* .184)—with the abovementioned caveat that *p*-values from exploratory analyses are not very meaningful.

Second, we only examined participants who did List 1 (the list with both “standard English” and “non-standard English”), excluding participants who did the other version of the survey in which all abstracts had “standard English”. This allows us to compare ratings within the same people as a function of language condition, but the limitation is that ratings are being made across different abstracts which probably had different intrinsic levels of scientific quality. We ran a new statistical model regressing scientific quality rating on language condition. (We did not include Abstract as a factor in this model: since there are only four abstracts, it probably does not have enough levels to be treated as a random effect, and also cannot be treated as a fixed effect since it is confounded with language condition, given that abstracts 1 and 3 only appeared in the “non-standard” condition and abstracts 2 and 4 only appeared in the “standard” condition.) Abstracts with “non-standard” English were given marginally lower ratings (*b* = −0.88, *z* = −1.85, *p* .064).

**Fig. 2.**
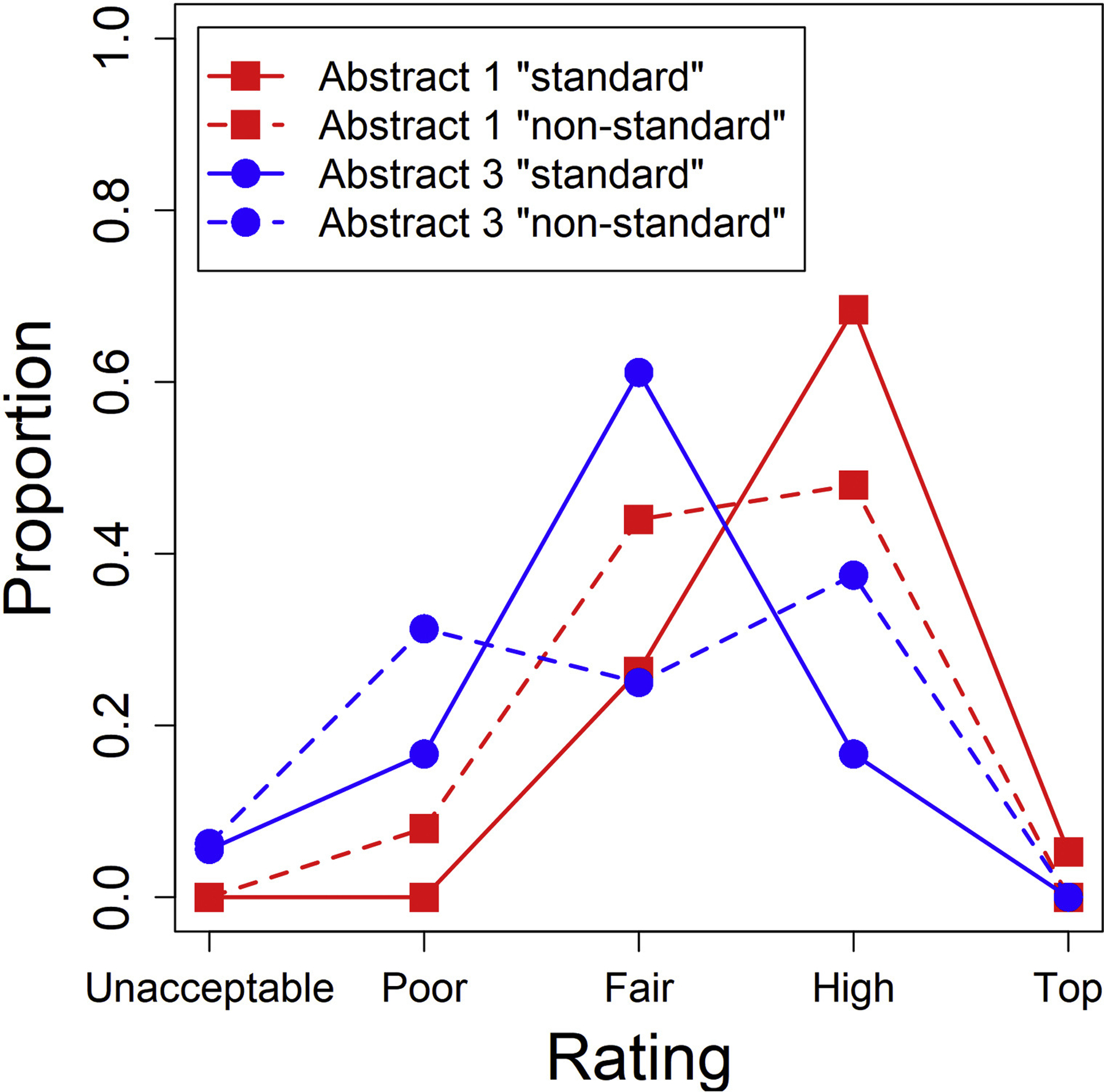
For each version of each abstract (only including abstracts 1 and 3), the proportion of participants giving each rating.

Third, we only examined abstracts #1 and #3, which were different across the two lists, and excluded observations from the other two abstracts (which were seen in the “standard English” condition by all participants). This allows us to compare ratings of the same abstracts as a function of language condition, but the limitation is that ratings are being made across different people, who may have different quality thresholds they use to make their evaluations. As shown in Fig. 2, participants rated abstract 3 lower than abstract 1 overall (i.e., abstract 3 had more “unacceptable” and “poor” ratings, and less “high quality” and “top quality” ratings). More importantly, abstract 1 clearly got better ratings (i.e., more “High-quality” and less “Poor” and “Fair”) when it appeared in “standard English” compared to when it appeared in “non-standard” English; abstract 3, on the other hand, did not show such a trend. The statistical model showed an effect of language condition that was in the same numerical direction as that shown in other analyses, but not statistically significant (*b* = −0.72, *z* = −1.26, *p* = .209).

**Fig. 3.**
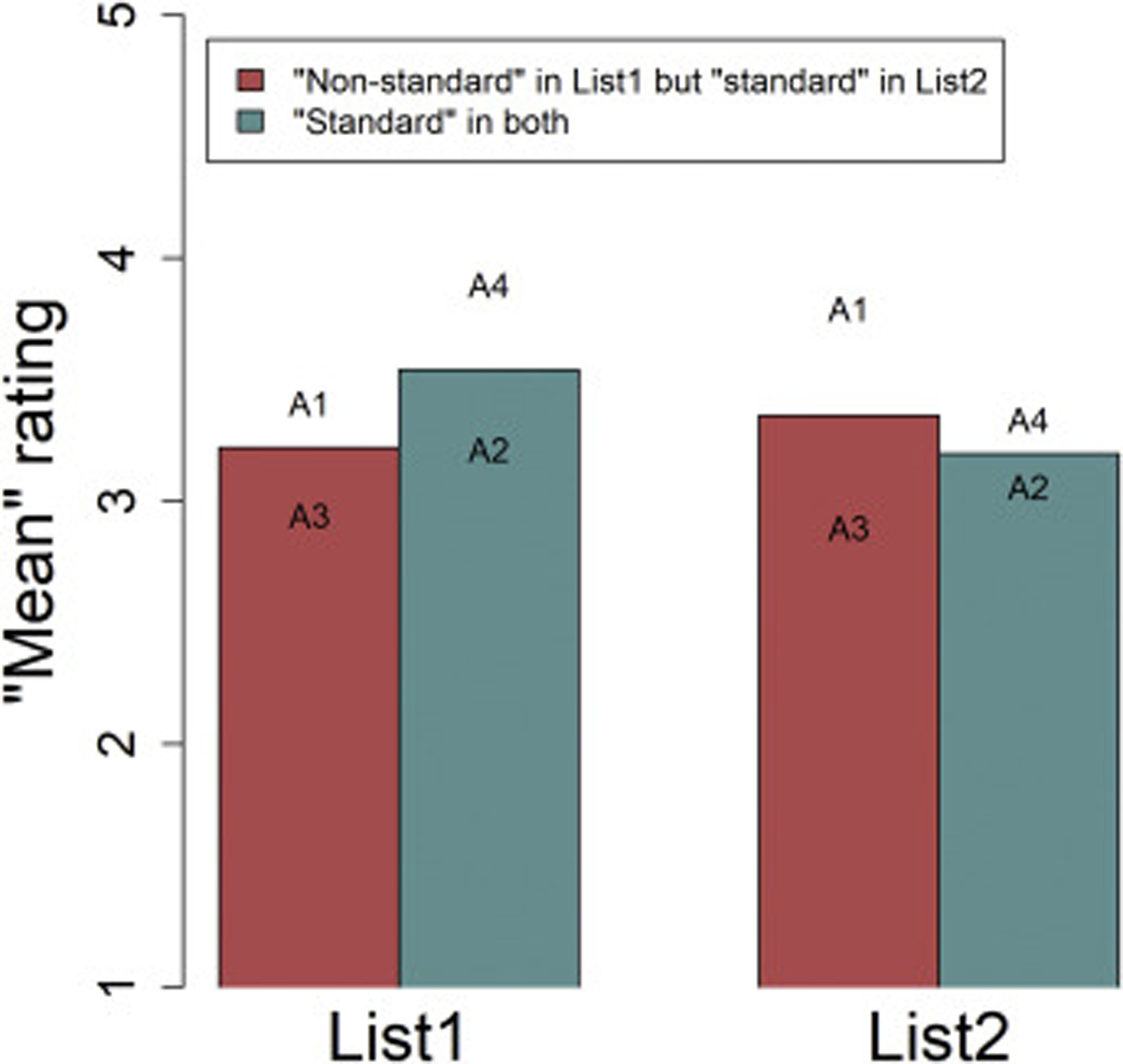
“Mean” ratings (based on treating the ordinal ratings as continuous data) for each type of abstract, in each list; see text for explanation. The text on the graph shows the “mean” rating for each individual abstract that was included in that abstract type (i.e., it can be seen that Abstract 1 received higher ratings than Abstract 3, and Abstract 4 received higher ratings than Abstract 2).

Fig. 3 displays what we consider the most appropriate analysis given the limitations of the present data. First, within List 1 (the version of the experiment in which two abstracts were in the “non-standard English” condition and two in the “standard English” condition), we compared the “non-standard English” abstracts to the “standard English” abstracts, just as was done in the second exploratory analysis above. As reported there and as shown on the left-hand side of Fig. 3, the “non-standard English” abstracts received worse ratings than the “standard English” ones. (For ease of exposition, Fig. 3 is showing mean ratings calculated by treating the ordinal rating scale as if it were continuous. That is not appropriate for ordinal data, but in the statistical model we do treat the data as ordinal; the graph is merely for visualization purposes.). As above, we cannot tell whether these abstracts were lower rated because of the “non-standard” language or because their content was intrinsically worse than that of the other two abstracts. In List 2, however, all four abstracts were written following a “standard English” conventions. Therefore, if abstracts 1 and 3 were inherently worse than abstracts 2 and 4, they should have been lower rated in List 2, just as they were in List 1. However, the graph below reveals that this is not the case; they were not substantially worse than the other two abstracts if we look at them in List 2. Thus, it is likely that the reason for their worse ratings in List 1 was the language, not the content. A statistical model testing the interaction between abstract type (abstracts 1 and 3, which differ between lists, vs. abstracts 2 and 4, which are the same across lists) and list was consistent with this observation. Within List 1, there was a nominally marginal (but keep in mind the abovementioned caveat about interpreting p-values in an exploratory analysis) difference between the two groups of abstracts, with abstracts 2 and 4 (the “standard English” ones) having higher ratings (*b* = 0.95, *z* = 1.94, *p* = .052) than abstracts 1 and 3 (the ones that had “non-standard English” in this list). Yet the concomitant difference between abstract types in List 2, compared to that difference in List 1, was smaller (interaction *b* = −1.43, *z* = −2.13, *p* = .033). In List 2, the difference between those abstracts was not significant (*b* = −0.49, *z* = −1.06, *p* = .290) and was in the opposite direction as the difference in List 1.

## 6. Discussion

In the present study, scholars evaluated the scientific quality of abstracts that were written in English that either conformed or did not conform to international standards of academic English. We found suggestive evidence that abstracts with international academic-standard English may tend to be judged as having higher scientific quality than abstracts with identical scientific content but English that does not meet international standards of academic English. While the results are statistically inconclusive, they offer the first empirical and experiment-based demonstration (to our knowledge) that scholars may exercise linguistic bias when reviewing academic writing. These results are potential evidence for one kind of linguistic injustice that may be at play for academic publishing. They highlight the need for further research on this topic.

One limitation of this study is that is used survey responses rather than real-life reviews. While the respondents were likely experienced reviewers (most were post-docs, lecturers, or professors), they were conducting the reviews in a different context than they would for real conference or journal reviewing. Therefore, even though our results suggest that linguistic bias may have occurred in this sample, we cannot rule out the possibility that reviewers may be less biased when they review for real conferences and journals. We find this unlikely, however, given that implicit biases are probably not subject to conscious control. (Indeed, if people actually are able to “turn off” their biases as this hypothetical argument would suggest, then that would mean that further research into linguistic injustice bias would be valuable, as calling more attention to the issue would empower people to be aware of and control their biases.) Furthermore, if reviewers show evidence of linguistic bias in an online survey, it would be likely that such bias may also appear in conference or journal reviews; while one might argue that reviews for real conferences and journals are done more diligently than reviews in this survey were, this point of view seems idealistic, given most scholars’ experience receiving cursory reviews (particularly for conferences, where a single reviewer may review a large number of abstracts in a short time). Nevertheless, future research in a more ecologically valid context would be valuable. For example, submitting abstracts for real review to an actual conference (in collaboration with the conference organizers) would yield more realistic data, and such collaborations with conference organizers have proven fruitful in the past (e.g., Roberts, Cuskley, Politzer-Ahles, & Verhoef, 2020; Roberts & Verhoef, 2016).

Another limitation has to do with the data. As described above, the sample ended up being much smaller than expected, because of high participant attrition. Furthermore, because of an error in the survey design, the study was not run in a full Latin square design as intended. Therefore, the statistical results are weak and exploratory. We opted not to attempt to re-run the study online with a proper Latin square, given the ecological validity limitation mentioned above. We felt that if this study is run again, it would be more valuable to do so with real conference reviews rather than with a survey.

A third limitation relates to the comprehensibility of the abstracts. As discussed above, we intentionally manipulated the language in a way that we expected would make one version of the abstract clearly diverge from international standards of academic English, *but* which would not impair comprehensibility. However, we have not empirically tested this assumption; our survey did not include any comprehension check to confirm that readers did understand the “non-standard English” abstracts as well as the “standard English” ones. Future studies should attend to this issue (either by including comprehension checks within the experiment, or by pre-testing the materials on a separate group of participants to ensure comprehensibility).

Finally, the present study only examines one way in which linguistic injustice could manifest during the peer review process. There are other ways that language could bias the outcomes of peer review. For example, Paltridge (2015) describes how reviewers often express suggestions/directives in the form of indirect requests, which may be misinterpreted by authors. While such misinterpretation problems are not limited to authors from non-English linguistic backgrounds (as Hyland, 2016a, points out, any authors unfamiliar with academic register, including “native” English speakers, may struggle with such issues), if such misinterpretation problems turn out to be more pervasive among non-native speakers than native speakers then they could also contribute to linguistic injustice. This is just one example; surely there are many possible mechanisms by which linguistic injustice might occur, and the present paper only tested evidence for one of them. For any other potential mechanism, empirical evidence may be needed to test whether that mechanism really does contribute to linguistic injustice or not.

How do the findings of this study relate to the broader question of linguistic injustice? There are really two questions at play in the recent debate over linguistic injustice: whether it exists at all, and whether it is worth researching or talking about. The present study challenges the notion that linguistic injustice does not exist at all: even though the statistical results are inconclusive, they at least suggest that linguistic bias may be occurring. That notion, however, may well have been a straw-person. While Hyland’s (2016a) paper which originally kicked off this debate made the provocative claim that linguistic injustice is a “myth”, most subsequent papers have taken the more nuanced position that linguistic injustice, even if it exists, is not worth focusing attention on. One argument made along these lines is that effects of linguistic injustice may be minor compared to those of other forms of injustice such as geographic inequalities in access to research resources and training (see, e.g., Hultgren, 2019). Another is that focus on supposed “deficiencies” associated with non-native speakers may be unconstructive or even harmful, and may discourage scholars who work in languages other than their first language, whereas a better way to support such scholars would be to shift the paradigm of academic publishing away from one that assumes a “deficiency” model, and to persuade gatekeepers to reconsider their expectations of all writing to conform to a certain English standard (e.g., Hyland, 2016a; McKinley & Rose, 2018). Similar arguments have also been made on the topic of gender and language, where the solution to language-related gender bias is not to train women to adopt more typically “masculine” speech styles, but rather to train the rest of society not to police and be biased against women’s speech; this issue is discussed in detail in, e.g., Deborah Cameron’s well-read blog (see, e.g., Cameron, 2015, for an example).

While both of the abovementioned positions have merit, we believe linguistic injustice is a topic worthy of discussion - and, critically, action. Even if there are other injustices which have a bigger impact on academic publishing (and there certainly are; we agree with Hultgren [2019] and others on this point), that does not mean that there is no point in documenting and understanding smaller injustices. If this were true, then presumably only one topic area (i.e., whatever the biggest injustice in the world is) would ever merit study. As for the second point, we agree that the goal of researchers should not be to discourage non-native English-speaking scholars or to reinforce a notion that they have any kind of “deficiency”. To the contrary, we believe that identifying and understanding the systematic challenges put up by the publishing process (including linguistic bias and others; see Politzer-Ahles et al., 2016, and Subtirelu, 2013, 2016) can both help the field to develop tools to address these challenges (e.g., to reduce reviewers’ biases—but that probably cannot be accomplished until those biases are acknowledged) and empower scholars working in non-native languages by revealing that some challenges they face are a result of systemic problems in the field and are *not* a result of the scholars’ own deficiencies.

## Supplementary Material

abstract_ratings_wide.csv

abstract_ratings_long.csv

Conference+Abstract+Review+Survey_July+7%2C+2019_16.48.csv

r_analysis.txt

## Figures and Tables

**Table 1:** Studies published since 2016 that make claims about whether or not linguistic injustice occurs in academic publishing. The summaries given here are necessarily oversimplified, given space constraints. The judgment of whether or not the study supports the idea that linguistic injustice exists is also necessarily simplistic (and reflects our own judgment of the study’s conclusion, not necessarily what the authors of a given study state), as it is too reductive to present this as a “yes or no” debate, and many of these studies specifically conclude that the reality is more complicated than that.

Paper	Method and finding	Linguistic injustice?
Botelho de Magalhães, Cotterall, and Mideros (2019)	Case studies of two scholars suggested that, while they were successful in developing their writing skills, it was challenging	Maybe
Hanauer, Sheridan, and Englander (2019)	In a survey, scholars self-reported that they felt greater burden when doing scientific writing in a second language (English) than in their first language	Yes
Langum and Sullivan (2017)	Analysis of Swedish-speaking doctoral students’ narratives showed that they perceive themselves to be deficient in not only English academic writing, but also in Swedish academic writing	No
McDowell and Liardét (2018)	Interviews and surveys with native Japanese-speaking scholars suggested that publishing in English, while more rewarding than publishing in Japanese, was also more challenging	Maybe
McKinley and Rose (2018)	Analysis of journals’ guidelines for authors showed that journal guidelines mandate a certain standard of English	Yes
Mirhosseini and Shafiee (2019)	Interviews with scholars suggested that publishing in English is more difficult than publishing in their native language (Farsi), and that the pressure to publish in English marginalizes the development of Farsi-language scholarship	Yes
Shvidko and Atkinson (2018)	Case studies of six early-career scholars showed that the non-native English speakers in the sample did not feel disadvantaged relative to the native English speakers	No
Soler and Cooper (2019)	Discourse analysis of e-mails from predatory publishers was consistent with speculation that the features of these e-mails may be more likely to trick non-native English speakers than native English speakers	Maybe
Strauss (2019)	Interviews with reviewers suggested that reviewers were biased against English that did not conform to their standard of “native-like” English	Yes
Yen and Hung (2019)	Analysis of publication patterns showed that English native speakers are overrepresented among authors, although language did not explain all of the differences	Maybe
Yotimart & Hashima Abd. Aziz (2017)	Analysis of interviews with eight scholars suggested that some of the non-native English speakers feel disadvantaged, although some of this may be attributable to non-linguistic factors	Maybe

## Footnotes

1 Pre-registration is a technique to increase reliability of and confidence in experimental findings (Nosek, Ebersole, DeHaven, & Mellor, 2017; Wagenmakers, Wetzels, Borsboom, van der Maas, & Kievit, 2012). It is meant to address the issue of flexibility in data analysis. There are many choices that can be made during data analysis, and it has long been known that trying out many different analyses, and selectively reporting just the ones that yielded the most attractive results, increases the likelihood of publishing implausible or un-replicable findings (Simmons, Nelson, & Simonsohn, 2011). This is the case even when the analysis choices look justifiable after the fact (Gelman & Loken, 2013). The best way to address this issue is to make all analysis decisions before having looked at the results, and keep a record of these decisions. This record is a pre-registration: a plan for how the data will be analyzed. Even if a pre-registration is not followed exactly, the pre-registration record makes it easier to distinguish between analyses that were planned ahead of time (and which can thus be considered *confirmatory*, offering evidence for a hypothesis) versus analyses or analysis steps that were only added after the data had been seen (and which thus must be considered *exploratory*, offering new hypotheses that still need to be tested in future research).

## Appendix A. “native-like English” version of Abstract #1

### Rationale

While much research has focused on first language acquisition of tone, there is little study of the development of tone in bilingual children whose dominant language is non-tonal and who are exposed to a tone language in school. To address this gap, we present the first empirical study documenting the characteristics of Urdu-Cantonese bilingual children’s production of Cantonese lexical tones.

### Methods

Twenty-one Urdu-Cantonese bilingual children aged from four to six years old and 20 monolingual Cantonese-speaking age-matched controls participated in the study. Tone production in monosyllabic and disyllabic words was examined by a picture-naming task. Tone productions were evaluated both acoustically and perceptually, with special emphases on tone height and tonal separation.

### Results

Acoustic and perceptual analyses indicated that the bilinguals exhibited an overall upshift of tone levels, and merging of T2/5, T3/6 and T4/6 relative to their monolingual Cantonese age peers. The findings indicated that despite being exposed to Cantonese from birth, these typically developing bilinguals exhibit noticeable problems with tone height of all 6 tones, and tonal separation of T2/T5, T3/T6, T4/T6 in their production.

### Discussion

The findings are useful for clinicians and educators working with bilingual children from the Pakistani heritage community in Hong Kong. This study provides evidence for Cantonese tones that are vulnerable for even typically developing bilinguals in this minority group. The results also lay a solid foundation for further studies examining lexical tone perception in these bilinguals.

## Appendix B. “Non-native-like English” version of Abstract #1

### Rationale

While much of the research focused on first language acquisition of tone, there is little study of development of the tone in bilingual child whose dominant language is non-tonal and who are exposed to tone language in school. To address this gap, we present the first empirical study documenting the characteristics about Urdu-Cantonese bilingual children’s producing of Cantonese lexical tones.

### Methods

Twenty-one Urdu-Cantonese bilingual children between 4 and 6 years old, and 20 children of Cantonese monolingual native speakers of same age range, participated in the study. They completed picture naming task on monosyllables and disyllables. Their accurate production was evaluated on two aspects: acoustic measurement with Praat, and the perceptual judgment by native speakers. Measurement focused on the tone height and tone separation.

### Results

Based on the acoustic measurement and perceptual test, the bilingual children have a higher tone than the Cantonese monolinguals. Also, their T2/T5, T3/T6, and T4/T6 categories were more merged than monolinguals categories. The findings indicated that even the bilinguals began to expose to Cantonese from birth, they showed obvious problems about the tone height of the six tones and the distinction between some pairs, when compared with the monolingual.

### Discussion

The finding is useful to the clinicians and educational workers for Pakistan heritage community in Hong Kong. It suggests that even for the typically developing bilinguals in the minority Pakistani heritage community there are still difficulties in learning Cantonese tones. This outcome will become the foundation for the further studies on the tonal process of the bilinguals.
